# Insight into the Role of Angiopoietins in Ageing-Associated Diseases

**DOI:** 10.3390/cells9122636

**Published:** 2020-12-08

**Authors:** Shin-ichiro Hayashi, Hiromi Rakugi, Ryuichi Morishita

**Affiliations:** 1 Department of Clinical Gene Therapy, Center of Medical Innovation and Translational Research, Osaka University Graduate School of Medicine, Suita, Osaka 565-0871, Japan; 2 Department of Geriatric and General Medicine, Osaka University Graduate School of Medicine, Suita, Osaka 565-0871, Japan; rakugi@geriat.med.osaka-u.ac.jp

**Keywords:** angiopoietin, ageing, ageing-associated diseases, endothelial cells, vascular function

## Abstract

Angiopoietin (Ang) and its receptor, TIE signaling, contribute to the development and maturation of embryonic vasculature as well as vascular remodeling and permeability in adult tissues. Targeting both this signaling pathway and the major pathway with vascular endothelial growth factor (VEGF) is expected to permit clinical applications, especially in antiangiogenic therapies against tumors. Several drugs targeting the Ang-TIE signaling pathway in cancer patients are under clinical development. Similar to how cancer increases with age, unsuitable angiogenesis or endothelial dysfunction is often seen in other ageing-associated diseases (AADs) such as atherosclerosis, Alzheimer’s disease, type 2 diabetes, chronic kidney disease and cardiovascular diseases. Thus, the Ang-TIE pathway is a possible molecular target for AAD therapy. In this review, we focus on the potential role of the Ang-TIE signaling pathway in AADs, especially non-cancer-related AADs. We also suggest translational insights and future clinical applications of this pathway in those AADs.

## 1. Introduction

The ageing population is growing rapidly in both developing and developed countries and is projected to reach more than 2.1 billion people by 2050 [1]. During the ageing process, humans face an increased risk of ageing-associated diseases (AADs), such as atherosclerosis, Alzheimer’s disease, type 2 diabetes, chronic kidney disease, cardiovascular diseases, chronic obstructive pulmonary disease and cancer [2]. Studies suggest that cellular ageing is the main contributor to these diseases and its biological mechanism involves genomic instability, epigenetic defects, dysregulation of metabolic pathways, increased cell senescence, impaired cell regeneration, increased reactive oxygen species by mitochondria and loss of proteostasis [3,4,5,6]. Other studies reported that cellular and the ensuing tissue dysfunctions in AADs are not only observed in each of their primary organs, but in other organs as well. For example, pathological organ-to-organ networks have been detected in heart disease, featuring chronic kidney disease [7,8]. This prompts the question of whether these organ-to-organ biological communications, predominantly within the circulatory system, correlate with multiple organ dysfunctions in AADs.

**Figure 1:**
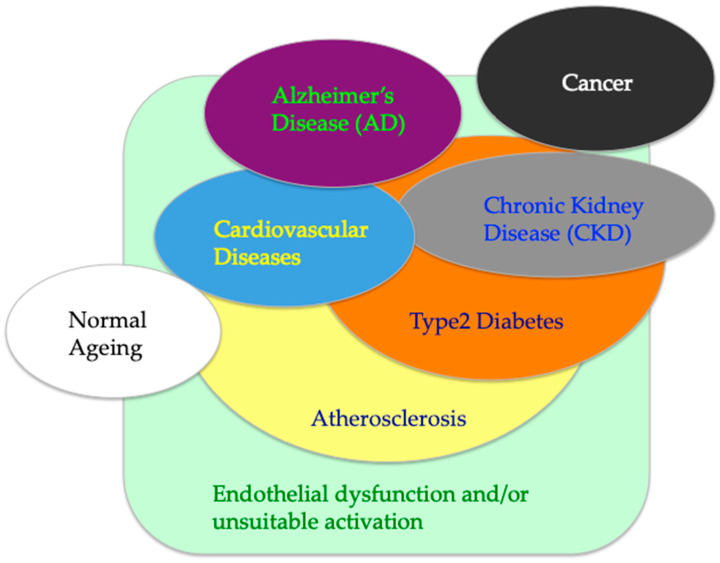
Correlation between vascular endothelial cells (ECs) and ageing-associated diseases (AADs). The schema illustrates the hypothesis that endothelial dysfunction and/or unsuitable activation appear as the forerunners of AADs, such as atherosclerosis, type 2 diabetes, chronic kidney disease (CKD), cardiovascular diseases, Alzheimer’s disease (AD) and cancer. Normal ageing also correlates with functional and phenotypic changes of ECs.

One of the crucial components of the circulatory system is vascular endothelial cells (ECs), which form a single-layer endothelium lining along the inner surface of the blood vessels throughout the vascular system. The endothelium serves a variety of functions in regulating local vascular inflammation, hemostasis, thrombolysis, proliferation of vascular smooth muscle cells (SMCs), vasoconstriction, vasodilation, angiogenesis and tissue regeneration; it also functions as a barrier between the vessels and tissues [9,10,11,12]. For the last few decades, families of endothelial cell-specific receptor tyrosine kinases and their ligands/growth factors—involving vascular endothelial growth factors (VEGFs), angiopoietin (Ang), and ephrin—have been identified as playing major roles in vascular development, remodeling and regeneration [9,13,14,15,16]. Bio-drugs targeting VEGFs and their receptors VEGFR signaling have been developed for clinical use, particularly in terms of antiangiogenic therapies against various types of tumor growth and metastasis [16]. Currently, new drugs targeting Ang and its receptor, TIE signaling, are being developed to treat cancer and aging-associated eye diseases [17,18], as this pathway plays a unique role in regulating vascular stability, remodeling, and angiogenesis [13,14]. Given the close relationship between AADs, the circulatory system and ECs (Figure 1), the vascular Ang-TIE pathway is a promising target for drug therapies treating not only cancer but numerous other AADs.

## 2. Ang-TIE Pathway in Vascular Endothelial Cells

**Figure 2:**
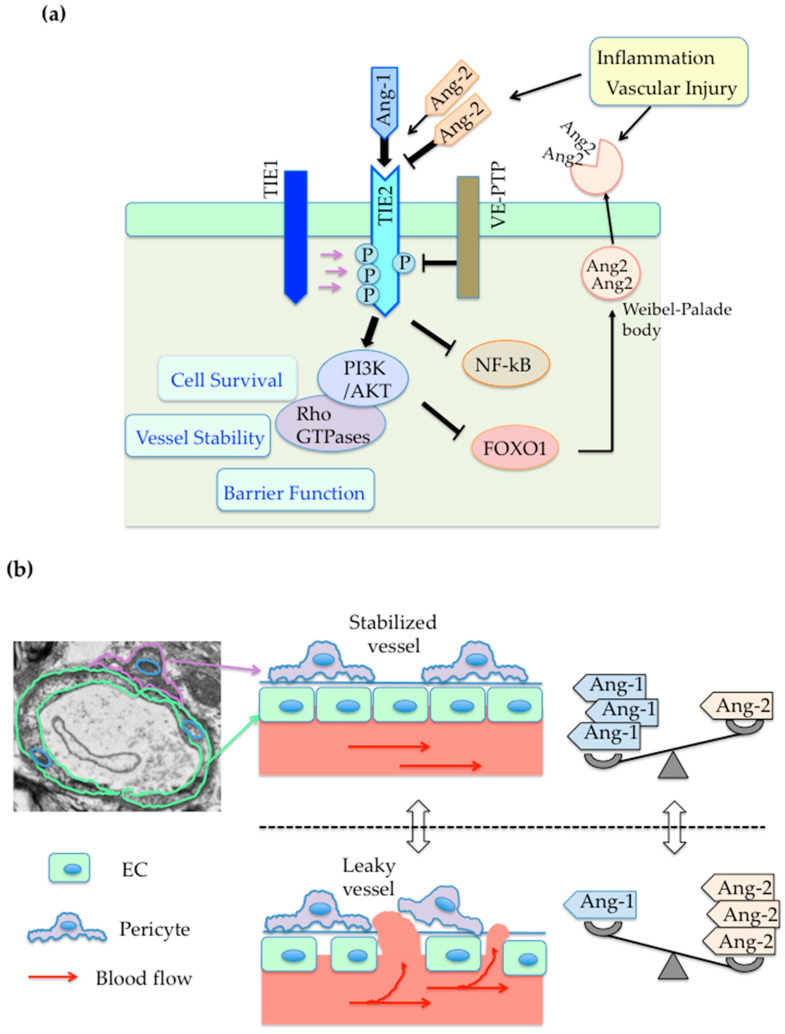
Angiopoietin–TIE signaling pathway in the vascular endothelium. (**a**) The schema indicates Ang-TIE signaling in vascular ECs. Ang1 promotes endothelial cell survival, vessel stability and barrier function via activation of signaling receptor TIE2 and its downstream targets, PI3K/AKT and Rho family GTPases. Ang1-TIE2-PI3K/AKT signaling inhibits NF-kB, FOXO 1 and FOXO 1-induced Ang2. Ang2 is a weak agonist or antagonist which modulates Ang1-TIE2 pathway. Upon inflammation and vascular injury, Ang2, stored in Weibel-Palade bodies, are released in circulation, antagonizes Ang1-TIE2 signaling and then increases FOXO1 and Ang2. Unlike signaling receptor TIE2, TIE1 is an orphan receptor homolog of TIE2, and mostly does not react to ligands. In some cases, TIE2 signaling is stimulated by phosphorylation due in part to the TIE receptor complex with TIE1. TIE2 activation is also achieved by the inhibition of a VE-PTP, which dephosphorylates and deactivates TIE2. Note that ‘P’ next to the intracellular part of TIE2 indicates phosphorylation. (**b**) The left upper panel shows transmission EM images with schematic indication of vascular ECs and pericytes. The right upper panel indicates that abundant Ang1 enhances endothelial barrier function and stabilizes vessels. In contrast, abundant Ang2 decreases endothelial barrier function and increases vascular permeability, leading to the formation of leaky vessels (Right lower panel).

The Ang ligands and their TIE receptors were discovered as essential molecules for cardiovascular development and adult vascular remodeling [13,14,19,20,21,22,23]. Angiopoietin-1 (Ang1), produced by pericytes or mesenchyme cells, acts as a paracrine ligand and a strong agonist for endothelial receptor tyrosine kinase TIE2. Ang1 activates signaling receptor TIE2, and promotes vessel stability, endothelial cell survival and barrier function via downstream targets of TIE2, including PI3 kinase/AKT, Rho family GTPases, actin-myosin cytoskeletons or VE-cadherin [14,21,22,24,25,26,27] (Figure 2a,b). Ang1-TIE2-AKT signaling also suppresses inflammation-associated molecules, such as NF-kB, vascular cell adhesion molecule 1(VCAM-1), intercellular adhesion molecule 1 (ICAM-1), Forkhead box protein O1 (FOXO 1) and another ligand for TIE2, angiopoietin-2 (Ang2) [28,29,30,31] (Figure 2a). In contrast, Ang2 is preferentially expressed by ECs and stored in Weibel-Palade bodies for its future release in blood circulation [32]. Ang2 acts as a conditional weak agonist or antagonist which inhibits the Ang1-TIE2 pathway [33]. Upon inflammation, Ang2 antagonizes TIE2, and thereby increases adult vascular leakage and instability [14,23,34] (Figure 2a,b). Ang2 also increases FOXO1 transcription and Ang2 reproduction in ECs [31,34]. Unlike signaling receptor TIE2, TIE1 is an orphan receptor homolog of TIE2. TIE1 mostly does not react to ligand stimulation, whereas TIE1 may modulate TIE2 signaling via its interaction with TIE2 at EC-EC junctions [20,35] (Figure 2a). Notably, TIE2 activation is also achieved by the inhibition of a transmembrane vascular endothelial protein tyrosine phosphate (VE-PTP) in EC-EC junctions, which dephosphorylates and deactivates TIE2 [27,36] (Figure 2a). Other transmembrane receptors, integrins, may have signaling interactions with the Ang-TIE pathway [37], while these interactions are still under investigation.

## 3. Ang-TIE Pathway in Atherosclerosis

Atherosclerosis is a slowly progressing AAD featuring lesion formation and luminal narrowing of the arteries, which gives rise to ischemic cardiovascular disease and multiple organ dysfunction via blood flow obstruction [38]. At the early stage of atherosclerosis, its underlying initiation mechanism involves endothelial dysfunction with senescent, proinflammatory and apoptotic phenotypes; these are commonly observed at arterial bifurcations with low laminar flow [39,40,41]. Through its progression, atherosclerosis plaques are gradually formed in the inner layer of arterial walls. Once plaque rupture and thrombosis occur near to the intimal lesion of the diseased arteries, this chronic AAD provokes life-threatening acute coronary syndrome, myocardial infarction or stroke [38,42].

Recent studies suggested that the Ang-TIE pathway may correlate with the initiation mechanism of atherosclerosis. For instance, atherogenic low laminar flow increased Ang2 expression in mouse aorta and ECs [43]. Conversely, protective high shear stress suppressed Ang2 expression in ECs [43]. Given the role of Ang2 as an antagonist for the Ang1-TIE2 pathway under inflammatory conditions [14,23,34], these data suggest that disturbed laminar flow may increase vascular inflammation and instability in part via the upregulation of Ang2. It is also noteworthy that atherogenic low laminar flow increased the expression of an orphan receptor TIE1 as well as several key molecules for atherogenesis, VCAM-1 and ICAM-1 [44,45]. Moreover, mice with TIE1 deletion showed a reduced number of atherosclerotic plaques [45]. These findings may be explained by the possible mechanism that TIE1 reduces the ability of Ang1 to activate TIE2 for vascular protection [35].

Another study showed that Ang2 blocking agents reduced both plasma triglycerides and early plaque formation in a murine model of hypercholesterolemia-induced atherosclerosis [46]. In addition, however, Ang2 blocking agents had no adverse effect on pre-existing atherosclerosis, suggesting that Ang2-TIE2 signaling actions may be limited to the early stages of atherosclerosis. In one study, intracoronary administration of Ang1 via an adenoviral vector protected against the development of cardiac allograft arteriosclerosis in rat-transplanted hearts [47]. This protective effect seems to involve Ang1-mediated anti-inflammatory properties and a reduction in plasma Ang2.

Altogether, these findings suggest that inhibiting Ang2-TIE2 or stimulating Ang1-TIE2 may have therapeutic effects on local vascular inflammation and plaque stability in the early phase of atherosclerosis. Furthermore, an orphan receptor TIE1 seems to modulate the activities of signaling receptor TIE2, and thereby create the conditions for vascular inflammation in atherosclerosis, whereas its role remains elusive.

## 4. Ang-TIE Pathway in Ischemic Heart Disease, Stroke, and Heart Failure

Cardiovascular diseases remain a leading cause of death worldwide; they include several critical AADs, such as ischemic heart disease, heart failure and ischemic stroke. Many ischemic heart diseases and strokes are related to ageing-associated vascular disorders, including the formation of atherosclerosis plaques, narrowing luminal arteries, and increasing the risk of plaque rupture and thrombosis. If a ruptured plaque with thrombus interrupts blood supply to the heart muscle or brain tissue, the cardiac myocytes or brain cells will begin to die, leading to acute coronary syndrome/myocardial infarction or ischemic stroke, respectively. Several reports have indicated that there is an association between ischemic cardiovascular disease and the Ang-TIE pathway.

A recent clinical trial demonstrated that high levels of Ang2 were an independent predictor of mortality in patients with acute myocardial infarction; it also suggested that an increase in plasma Ang2 levels might partly reflect persistent endothelial damage in ischemic heart conditions [48]. In support of this, an in vivo study using a murine model of myocardial infarction or ischemia/reperfusion injury reported that Ang2 was highly expressed in the ECs present at the infarcted border zone. Furthermore, these cells promoted abnormal remodeling, inflammation and cardiac hypoxia by inhibiting Ang1-TIE2 signaling [49]. Notably, after myocardial ischemia, an Ang2 blockade improved this pathological remodeling [49].

In a murine model of stroke, overexpression of Ang2 resulted in increased infarct sizes and vessel permeability in the subjects’ brain tissues [50]. In contrast, Ang1-TIE2 signaling activation via inhibiting the vascular endothelial protein tyrosine phosphatase, VE-PEP [27], alleviated Ang2-induced increases in vessel permeability and infarct sizes [50]. Supporting these findings, two studies using human bio-banked blood samples or brain sections from autopsy specimens revealed that Ang2 expression levels were significantly upregulated in stroke patients, whereas those of Ang1 were decreased in the group with ischemic stroke [50,51]. In support of this, studies in murine ischemic stroke models showed that pharmacological- or stem cell-induced Ang1 upregulation significantly improved vasogenic edema and outcomes of stroke [52,53].

Heart failure (HF) is a chronic and progressive AAD that represents insufficient cardiac output due to structural or functional impairment of cardiac constriction or relaxation. Recently, along with classical HF with reduced ejection fraction (HFrEF), a new type of HF with preserved ejection fraction (HFpEF) has emerged among the ageing population [54]. HFpEF is characterized as a contractile dysfunction with normal EF; it represents endothelial dysfunction, which is induced by comorbidities such as ageing, hypertension, diabetes, and obesity. In one recent study, an analysis of blood samples from HFpEF patients revealed that Ang2 was one of the predictive biomarkers for HF-related hospital admission [55]. This indicates that Ang2 levels may reflect endothelial damage and the severity of HFpEF. Similarly, another recent study using integrated electric health records, clinical blood samples, and proteomics analysis demonstrated that Ang2 and thrombospondin-2 robustly predicted acute HF [56].

Collectively, these findings suggest the possibility that Ang2 secreted from ECs may be a potential biomarker that could aid in the diagnosis and risk assessment of ischemic cardiovascular diseases and HF. They also imply that stimulating Ang1-TIE2 or blocking Ang2-TIE2 signaling may govern proper tissue angiogenesis in ischemic cardiovascular diseases and HF.

## 5. Ang-TIE Pathway in Peripheral Arterial Disease

Similar to ischemic heart diseases and stroke, peripheral artery disease (PAD) is also implicated in ageing-associated atherosclerosis [57]. The severe form of PAD, known as a critical limb ischemia (CLI), often represents claudication, rest pain and unhealing ulcers, ultimately resulting in limb amputation. To avoid these conditions, in addition to the general treatments for symptomatic PAD or CLI (e.g., anti-platelet regimens and common medications for atherosclerosis, exercise therapy, angioplasty, stenting or bypass surgery), angiogenic growth factors have been highlighted as new treatment options, which might promote therapeutic angiogenesis and arteriogenesis (i.e., the growth of existing collateral vessels) in ischemic limbs [58].

Despite the initial promise of approaches using the potent angiogenic growth factor VEGF in the setting of CLI, there remains concerns about undesirable side effects, such as VEGF-induced leaky neovessels, angioma formation, or recruitment of inflammatory cells in ischemic tissues [59,60]. However, Ang-1 combined with VEGF induced non-leaky neovessels [22], and indeed, a preclinical report on a murine model of limb ischemia showed that VEGF-Ang1 chimeric gene or protein may promote neovascularization, which represented less leakiness, less tissue inflammation, less angioma-like formation, and better perfusion recovery as compared with VEGF alone [61]. Consistently, an open-label phase 1b study in a small group of patients with severe CLI showed higher rates of ulcer healing and one-year amputation-free survival in patients intra-arterially administered autologous venous ECs and SMCs, which enhanced Ang1 and VEGF expression, respectively [62]. It is also noteworthy that plasma levels of VEGF and soluble TIE2 were significantly higher in patients with CLI compared with healthy control subjects, suggesting mechanistic crosstalk between VEGF-VEGFR signaling and Ang-TIE signaling in patients with CLI [63]. Notably, a recent experimental study showed that an increase in limb Tie2 expression by modulating microRNA-15a/-16 may improve tissue angiogenesis and perfusion after limb ischemia [64]. In addition, Ang1 production stimulated by hepatocyte growth factor may ameliorate PAD via stabilizing the neovessels [65,66]. In contrast to Ang1 or TIE2, however, the role of Ang2 in PAD or CLI still remains elusive.

## 6. Ang-TIE Pathway in Chronic Kidney Disease

Chronic kidney disease (CKD) is characterized by the gradual loss of kidney function and is highly associated with cardiovascular disease and its risk factors (i.e., diabetes and hypertension). CKD also represents a pathological connection with other AADs (Figure 1). Considering the close relationship between CKD and atherosclerosis or cardiovascular diseases [7], Ang-TIE signaling may play a role in CKD pathogenesis.

A cross-sectional cohort study revealed that increased plasma Ang2 levels were independently associated with a worsening of arterial stiffness, a cardiovascular risk, in patients with CKD [67]. In support of this, murine CKD models showed that Ang2 expression was significantly increased in the plasma and kidneys after partial nephrectomy or unilateral ureteral obstruction [67]. In contrast, a decrease in Ang1 expression was observed in the kidneys and aorta of these models. Moreover, blocking Ang2 decreased the expression of profibrotic and proinflammatory cytokines in the aorta of these mice [67], suggesting that increased plasma Ang2 in CKD seem to correlate with enhanced inflammatory and fibrotic signaling in ECs. Another cross-sectional and longitudinal observation study also showed that serum Ang2 levels increased in patients undergoing dialysis, whereas their Ang1 levels were decreased [68]. In addition, Ang2 levels normalized three months after kidney transplantation. Importantly, Ang2 levels in CKD patients significantly correlated with atherosclerotic scores of coronary heart disease and PAD [68]. One study reported consistent results indicating that Ang2 levels were higher in end-stage CKD patients on dialysis. Interestingly, elevated Ang2 levels are strong predictors of long-term mortality, independent of arterial stiffness or vascular calcification [69].

A few experimental studies focused on the role of the Ang-TIE pathway in early stages of CKD. Proteinuria is an early sign of kidney damage and thus a useful marker for detecting early-stage CKD. Notably, mice with podocyte-specific Ang2 overexpression induced proteinuria and apoptosis of the glomerular endothelia, indicating the possibility that Ang2 may worsen proteinuria in the initiation and progression of CKD [70].

These findings suggest the possibility that Ang2 may be a useful biomarker as well as therapeutic target for CKD. In addition, stimulating Ang1-TIE2 signaling may be utilized for CKD treatment via stabilization of ECs and reduction in local inflammation, although this will need further investigation.

## 7. Ang-TIE Pathway in Diabetic Vascular Complications

Type 2 diabetes is an AAD with chronic metabolic disorder characterized by hyperglycemia and insulin resistance, which leads to vascular complications such as cardiovascular comorbidities, nephropathy, retinopathy, neuropathy, and impaired wound healing [71]. Patients with type 2 diabetes are also at risk for developing Alzheimer’s disease [72]. As underlying initiation and progression mechanisms of these complications presumably involve endothelial dysfunction and unsuitable angiogenesis (Figure 1), vascular Ang-TIE signaling may have a role in the pathogenesis of diabetes. For instance, clinical data showed that plasma Ang2 and VEGF levels were elevated in patients with diabetes, and were also associated with indexes of endothelial damage/dysfunction [73]. In addition, increased Ang2 expression was detected in both experimental models and patients with diabetic retinopathy [74,75].

**Figure 3:**
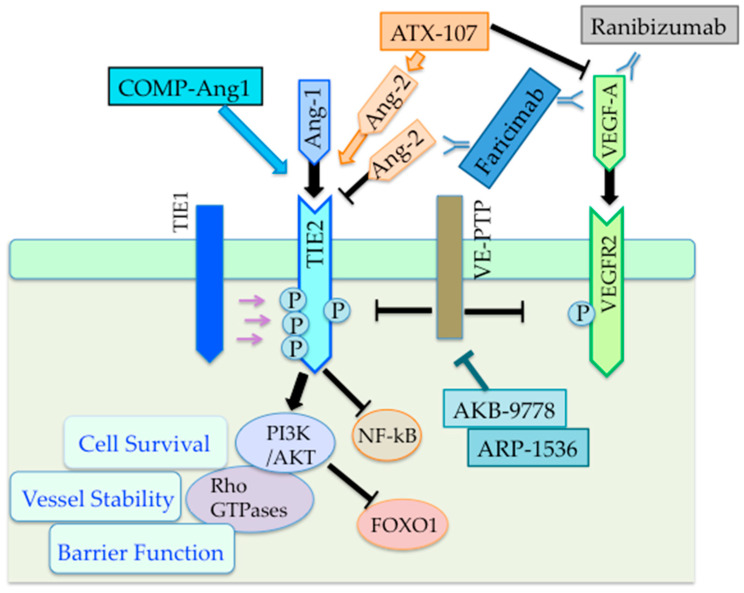
Ang-TIE therapeutics against diabetic retinal diseases. The schema indicates possible Ang-TIE targeting drugs for diabetic retinal diseases and their signaling targets in ECs. Faricimab, a bispecific antibody that targets both Ang2 and VEGF-A, is in phase 3 clinical trials for diabetic retinal diseases, such as diabetic macular edema (DME). VE-PTP inhibitors, ARP-1536 and AKB-9778, which can activate the Ang1-TIE2 pathway, are under clinical development for DME. In addition, VE-PTP inhibition by those drugs deactivate VEGFR2. In a phase 2a clinical trial, AKB-9778 with ranibizumab, a recombinant VEGF-A antibody, resulted in significant reduction in DME. A collagen IV-derived peptide, ATX107, is under clinical development. ATX107 uniquely converted Ang2 into a TIE2 agonist, and thereby activating TIE2 signaling in experimental models. ATX107 also inhibited VEGF signaling. In experimental models of diabetes, soluble Ang1 variant, COMP-Ang1, protected retinal vascular structure, blood retinal barrier integrity, and visual dysfunction.

Diabetic retinopathy and subsequent diabetic macular edema (DME) are the most common complications of diabetes that lead to vision loss. Given that the mechanism involves retinal pericytes dropout, local ischemia, abnormal neovascularization, and excessive vascular leakage, proper regulation of retinal vascular stability and angiogenesis by Ang-TIE pathway may have therapeutic potentials for these complications. Studies showed that diabetic mice with heterozygous Ang2 deletion inhibited retinal pericytes loss and decreased the number of capillary segments [76], and intravitreal administration of recombinant Ang2 increased pericyte loss and vascular permeability [75,76]. Conversely, intravitreal injection of soluble Ang1 variant, cartilage oligomeric matrix protein-Ang1 (COMP-Ang1), protected retinal vascular structure, blood retinal barrier integrity, and visual dysfunction in a murine diabetic model [77,78] (Figure 3). Faricimab, a bispecific antibody that targets both Ang2 and VEGF, is in phase 3 trials for DME and age-related macular degeneration [18] (Figure 3). Currently, administration of VE-PTP inhibitors/Ang1-TIE2 activators (i.e., ARP-1536 and AKB-9778) is also under clinical development for DME [18,36] (Figure 3). In contrast to the intravitreal delivery of ARP-1536, AKB-9778 can be subcutaneously injected [18]. In a phase 2a randomized clinical trial, the combined therapy of AKB-9778 with ranibizumab, a recombinant humanized monoclonal antibody fragment against VEGF-A, resulted in significantly greater reduction in DME compared with ranibizumab alone [79]. More recently, a collagen IV-derived peptide under clinical development, ATX107, uniquely converted Ang2 into a TIE2 agonist, thereby activating TIE2 signaling in experimental models [18] (Figure 3). Notably, in addition to TIE2 activation, ATX107 inhibited signaling through VEGFR2. At present, however, safety and efficacy issues of this compound remain to be proven.

As described in the CKD section above, diabetic nephropathy is the leading cause of CKD with renal failure. In experimental models of murine diabetes, Ang2 expression was upregulated in glomerular ECs at an early phase of diabetes [70], whereas glomerular Ang1 expression was decreased [80]. Furthermore, pharmacological blockade of both Ang2 and VEGF reduced pathological alterations in early diabetic nephropathy, including glomerular hypertrophy, hyperfiltration and albuminuria [81]. Podocyte-specific overexpression of *Ang1* gene reduced albuminuria and increased endothelial nitric oxide synthase [80]. Moreover, genetic deletion of VE-PTP that restored TIE2 activation protected renal structure and function in a murine model of diabetic nephropathy [82].

A diabetic foot ulcer (DFU) is one of the serious complications of diabetes, and is often presented with wound infection, impaired wound healing, and PAD [83]. More than 40% of DFU patients are comorbid with PAD, and these patients have a higher amputation rate and a higher mortality rate [84]. The mechanism underlying diabetic unhealing ulcers reportedly involves endothelial dysfunction, persistent inflammation, insufficient tissue angiogenesis and impaired re-epithelialization [85]. In addition to the standard management of DFU (e.g., surgical debridement, wound-off-loading, infection control and vascular assessment) [86], human growth factors acting on vascular cells and/or epithelial cells, such as VEGF, fibroblast growth factor, platelet-derived growth factor and epidermal growth factor, have been investigated for the clinical treatment of DFU. The beneficial use of those growth factors in DFU, however, remains elusive [86]. In contrast, several studies have focused on Ang–TIE signaling in wound healing and on its application in DFU. A murine type 2 diabetes model showed sustained high Ang2 expression during wound healing, whereas TIE2 and VEGF-A expression levels were markedly reduced in wound tissues [87], indicating that Ang1-TIE2 and VEGF-VEGFR2 signaling may be insufficient during wound repair in diabetes. Consistent with this, a report showed that systemic and topical replenishing with COMP-Ang1 accelerated wound healing by enhancing angiogenesis and lymphangiogenesis in type 2 diabetic mice [88]. Recently, an Ang1-derived integrin-binding prosurvival peptide, QHREDGS, was shown to accelerate wound healing in diabetic mice via promoting re-epithelization or vascular endothelial cell survival [89,90].

Taken together, these findings suggest that stimulating Ang1-TIE2 or inhibiting Ang2-TIE2 signaling may have beneficial effects on vascular stability or suitable angiogenesis in diabetic vascular complications. Ang1-TIE2 stimulation combined with blocking VEGF-VEGFR2 may be especially useful for diabetic retinopathy and DME.

## 8. Ang-TIE Pathway in Alzheimer’s Disease

Alzheimer’s disease (AD) is a progressive neuronal disorder that causes dementia and death in elderly people. The typical features of AD involve accumulation of beta amyloid-containing plaques and neurofibrillary tangles in the diseased brain [91,92,93]. It has been suggested that more than 90% of AD are a non-genetic, sporadic form of the disease; furthermore, familial cases (5%) represent mutation in the amyloid precursor proteins presenilin1 and presenilin2 [94]. Recent studies have shown that the pathogenesis of AD is not limited to the neurons but also associated with the vascular system in the form of vascular risk factors and cardiovascular diseases [95,96,97]. Several studies have reported the possible correlation between AD and Ang-TIE signaling.

A recent report using data from Framigham Heart Study participants, magnetic resonance imaging (MRI) data and blood samples found that higher Ang2 levels in the blood were associated with decreased fractional anisotrophy in the white matter of the APOE-ε4 carrier [98,99]. This indicates that a higher level of Ang2 may correlate with a higher risk group in AD. Given that increased Ang2 expression correlates with endothelial damage and inflammation in ischemic cardiovascular disease, the findings in this study also suggest that higher Ang2 levels may indicate the existence of endothelial damage and inflammation in the early stages of AD. Another clinical study showed that serum Ang1 expression levels were higher in AD patients versus healthy controls [100]. In addition, Ang1 serum levels showed a significant correlation with cognitive status in all patients with AD, with mild cognitive impairment and in healthy controls. Although the results indicate that serum Ang1 may be used as a biomarker for AD, it is still unclear whether Ang1 has protective or worsening effects for AD. It is also uncertain whether neurovessels stabilized by Ang1 may impair the clearance of beta amyloid and tau in diseased brains.

## 9. Conclusions

This review highlights the potential roles and future applications of targeting the Ang–TIE signaling pathway in AADs involving atherosclerosis, cardiovascular diseases, chronic kidney disease, diabetic vascular complications and Alzheimer’s disease. Because of its unique functions on vascular ECs, modulating vessel stability, endothelial cell survival and its barrier function (Figure 2), the Ang–TIE signaling pathway could be an intriguing molecular target for numerous AADs with endothelial dysfunction and unsuitable angiogenesis (Figure 1). Currently, several pharmaceutical drugs targeting the key players of this pathway (i.e., Ang2, both Ang2 and VEGF, both Ang1 and Ang2, VE-PTP or TIE2) are under clinical development, especially for the treatment of human cancer or diabetic retinal diseases [17,18]. In addition, the expression levels of plasma Ang1 or Ang2 may be utilized as biomarkers for atherosclerosis, cardiovascular diseases, chronic kidney disease, diabetic vascular complications and Alzheimer’s disease. Future investigations in endothelial Ang-TIE signaling may also unravel the molecular mechanisms underlying multiple organ dysfunctions and multimorbidity in AADs [101]. Moreover, biologics targeting to Ang1-TIE2 pathway may be applied to the high fatality rate of the latest global crisis, COVID-19 infection and its comorbidities, involving acute respiratory distress syndrome, cardiovascular diseases, obesity and diabetes [102,103,104,105,106].

## Abbreviations

Ang	angiopoietin
VEGF	vascular endothelial growth factor
AADs	ageing-associated diseases
ECs	endothelial cells
SMCs	smooth muscle cells
Ang1	angiopoietin-1
VCAM-1	vascular cell adhesion molecule 1
FOXO 1	Forkhead box protein O1
Ang2	angiopoietin-2
VE-PEP	vascular endothelial protein tyrosine phosphate
HF	heart failure
HFrEF	heart failure with reduced ejection fraction
HFpEF	heart failure with preserved ejection fraction
PAD	peripheral artery disease
CLI	critical limb ischemia
CKD	chronic kidney disease
DME	diabetic macular edema
COMP	cartilage oligomeric matrix protein; angiopoietin-1
DFU	diabetic foot ulcer
AD	Alzheimer’s disease
MRI	magnetic resonance imaging

## References

[1] United Nations Department of Economic and Social Affairs, Population Division (2015). World Population Ageing 2015.

[2] (2019). Deaths: Leading Causes for 2017.

[3] Van Deursen J.M. (2014). The role of senescent cells in ageing. Nature.

[4] Barker D.J., Childs B.G., Durik M., Wijers M.E., Sieben C.J., Zhong J., Saltness R.A., Jeganathan K.B., Casaclang Verzosa G., Pezeshki A. (2016). Naturally occurring p16ink4a-positive cells shorten healthy lifespan. Nature.

[5] Childs B.J., Baker D.J., Wijshake T., Conover C.A., Campisi J., van Deursen J.M. (2016). Senescent intimal foam cells are deleterious at all stages of atherosclerosis. Science.

[6] Lopez-Otin C., Blasco M.A., Partridge L., Serrano M., Kroemer G. (2013). A comprehensive overview of cellular defects that occur during ageing. Cell.

[7] Go A.S., Chertow G.M., Fan D., McCulloch C.E., Hsu C. (2004). Chronic kidney disease and the risks of death, cardiovascular events, and hospitalization. N. Engl. J. Med..

[8] Warnner C., Lachin J.M., Inzucchi S.E., Fitchett D., Mattheus M., George J., Woerle H.J., Broedi U.C., von Eynatten M., Zinman B. (2018). EMPA-REG OUTCOME investigators. Empagliflozin and clinical outcomes in patients with type 2 diabetes mellitus, established cardiovascular disease, and chronic kidney disease. Circulation.

[9] Risau W. (1997). Mechanism of angiogenesis. Nature.

[10] Forkman J. (1995). Angiogenesis in cancer, vascular, rheumatoid and disease. Nat. Med..

[11] Kipshidze N., Dangas G., Tsapenko M., Moses J., Leon M.B., Kutryk M., Serruys P. (2004). Role of the endothelium in modulating neointimal formation: Vasculoprotective approaches to attenuate restenosis after percutaneous coronary interventions. J. Am. Coll. Cardiol..

[12] Asahara T., Isner J.M. (2000). Endothelial progenitor cells for vascular regeneration. J. Hematother. Stem Cell Res..

[13] Gale N.W., Yancopoulos G.D. (1999). Growth factors acting via endothelial cell-specific receptor tyrosine kinases: VEGFs, angiopoietins, and ephrins in vascular development. Genes Dev..

[14] Koh G.Y. (2013). Orchestral actions of angiopoietin-1 in vascular regeneration. Trends Mol. Med..

[15] Hayashi S., Asahara T., Masuda H., Isner J.M., Losordo D.W. (2005). Functional ephrin-B2 expression for promotive interaction between arterial and venous vessels in postnatal neovascularization. Circulation.

[16] Apte R.S., Chen D.S., Ferrara N. (2019). VEGF in signaling and disease: Beyond discovery and development. Cell.

[17] Fukumura D., Kloepper J., Amoozgar Z., Duda D.G., Jain J.K. (2018). Enhancing cancer immunotherapy using antiangiogenics: Opportunities and challenges. Nat. Rev. Clin. Oncol..

[18] Hussain R.M., Neiweem A.E., Kansara V., Harris A., Ciulla T.A. (2019). Tie-2/Angiopoietin pathway modulation as a therapeutic strategy for retinal disease. Expert Opin. Investig. Drugs.

[19] Dumont D.J., Yamaguchi T.P., Conlon R.A., Rossant J., Breitman M.L. (1992). Tek, a novel tyrosine kinase gene located on mouse chromosome 4, is expressed in endothelial cells and their presumptive precursors. Oncogene.

[20] Partanen J., Armstrong E., Mäkelä T.P., Korhonen J., Sandberg M., Renkonen R., Knuutila S., Huebner K., Alitalo K. (1992). A novel endothelial cell surface receptor tyrosine kinase with extracellular epidermal growth factor homology domains. Mol. Cell. Biol..

[21] Suri C., Jones P.F., Paten S., Bartunkova S., Maisonpierre P.C., Davis S., Sato T.N., Yancopoulos G.D. (1996). Requisite role of angiopoietin-1, a ligand for the TIE2 receptor, during embryonic angiogenesis. Cell.

[22] Thurston G., Rudge J.S., Isoffe E., Zhou H., Ross L., Croll S.D., Glazer N., Holash J., McDonald D.M., Yancopoulos G.D. (2000). Angiopoietin-1 protects the adult vasculature against plasma leakage. Nat. Med..

[23] Maisonpierre P.C., Suri C., Jones P.F., Bartunkova S., Wiegand S.J., Radziejewski C., Compton D., McClain J., Aldrich T.H., Papadopoulos N. (1997). Angiopoietin-2, a natural antagonist for Tie2 that disrupts in vivo angiogenesis. Science.

[24] Kim I., Kim H.G., So J.N., Kim J.H., Kwak H.J., Koh G.Y. (2000). Angiopoietin-1 regulates endothelial cell survival through the phosphatidylinositol 3′-kinase/Akt signal transduction pathway. Circ. Res..

[25] Mammoto T., Parikh S.M., Mammote A., Gallagher D., Chan B., Mostoslavsky G., Ingber D.E., Sukhatme V.P. (2007). Angiopoietin-1 requires p190 RhoGAP to protect against vascular leakage in vivo. J. Biol. Chem..

[26] David S., Ghosh C.C., Mukherjee A., Parikh S.M. (2011). Angiopoietin-1 requires IQ domain GTPase-activating protein 1 to activate Rac1 and promote endothelial barrier defence. Arterioscler. Thromb. Vasc. Biol..

[27] Frye M., Dierkes M., Küppers V., Vockel M., Tomm J., Zeushner D., Rossaint J., Zarbock A., Koh G.Y., Peters K. (2015). Interfering with VE-PTP stabilizes endothelial junctions in vivo via Tie-2 in the absence of VE-cadherin. J. Exp. Med..

[28] Hughes D.P., Marron M.B., Brindle P.J. (2003). The anti-inflammatory endothelial tyrosine kinase Tie2 interacts with a novel nuclear factor-kappaB inhibitor ABIN-2. Circ. Res..

[29] Kim I., Moon S.O., Park S.K., Chae S.W., Koh G.Y. (2003). Angiopoietin-1reduces VEGF-stimulated leukocyte adhesion to endothelial cells by reducing ICAM-1, VCAM-1, and E-selectin expression. Circ. Res..

[30] Daly C., Wong V., Burova E., Wei Y., Zabski S., Griffiths J., Lai K.M., Lin H.C., Ioffe E., Yancopoulos G.D. (2004). Angiopoietin-1 modulates endothelial cell function and gene expression via the transcription factor FKHR (FOXO1). Genes Dev..

[31] Scharpfenecker M., Fiedler U., Reiss Y., Augustin H.G. (2005). The Tie-2 ligand angiopoietin-2 destabilizes quiescent endothelium through an internal autocrine loop mechanism. J. Cell Sci..

[32] Fielder U., Scharpfenecker M., Koidl S., Hegen A., Grunow V., Schmidt J.M., Kriz W., Thurston G., Augustin H.G. (2004). The Tie-2 ligand angiopoietin-2 is stored in and rapidly released upon stimulation from endothelial cell Weibel-Palade bodies. Blood.

[33] Yuan H.T., Khankin E.V., Karumanchi S.A., Parikh S.M. (2009). Angiopoietin 2 is a partial agonist/antagonist of Tie2 signaling in endothelium. Mol. Cell. Biol..

[34] Benest A.V., Kruse K., Savant S., Thomas M., Laib A.M., Loos E.K., Fiedler U., Augustin H.G. (2003). Angiopoietin-2 is critical for cytokine-induced vascular leakage. PLoS ONE.

[35] Korhonen E.A., Lampinen A., Giri H., Anisimov A., Kim M., Allen B., Fang S., D’Amico G., Sipilä T.J., Lohela M. (2016). Tie1 controls angiopoietin function in vascular remodeling and inflammation. J. Clin. Investig..

[36] Shen J., Frye M., Lee B.L., Reinardy J.K., McClung J.M., Ding K., Kojima M., Xia H., Seisel C., e Silva R.L. (2014). Targeting VE-PTP activates TIE2 and stabilizes the ocular vasculature. J. Clin. Investig..

[37] Cascone I., Napione L., Maniero F., Serini G., Bussolino F. (2005). Stable interaction between α5β1 integrin and Tie2 tyrosine kinase receptor regulates endothelial cell response to Ang1. J. Cell Biol..

[38] Christian W., Heidi N. (2011). Atherosclerosis: Current pathogenesis and therapeutic options. Nat. Med..

[39] Gimbrone M.A., Garcia-Gardeña G. (2013). Vascular endothelium, hemodynamics, and the pathobiology of atherosclerosis. Cardiovasc. Pathol..

[40] Minamino T., Miyauchi H., Yoshida T., Ishida Y., Yoshida H., Komuro I. (2002). Endothelial cell senescence in human atherosclerosis: Role of telomere in endothelial dysfunction. Circulation.

[41] Dimmer S., Haendeler J., Rippmann V., Nehls M., Zeiher A.M. (1996). Shear stress inhibits apoptosis of human endothelial cells. FEBS Lett..

[42] Libby P. (2013). Mechanism of acute coronary syndromes and their implications for therapy. N. Eng. J. Med..

[43] Tiressel S.L., Huang R.P., Tomsen N., Jo H. (2007). Laminar shear stress inhibits tubule formation and migration of endothelial cells by an angiopoietin-2 dependent mechanism. Arterioscler. Thromb. Vasc. Biol..

[44] Porat R., Grunewald M., Globerman A., Itin A., Barshtein G., Alhonen L., Alitalo K., Keshet E. (2004). Specific induction of tie1 promoter by disturbed flow in atherosclerosis-prone vascular niches and flow-obstructing pathologies. Circ. Res..

[45] Woo K.V., Qu X., Babaev V.R., Linton M.F., Cuzman R.J., Fazio S., Baldwin H.S. (2011). Tie1 attenuation reduces murine atherosclerosis in a dose-dependent and shear stress-specific manner. J. Clin. Investig..

[46] Theelen T.L., Lappalainen J.P., Sluimer J.C., Gurzeler E., Celeutjens J.P., Gijbels M.J., Biessen E.A., Daemen M.J.A.P., Alitaro K., Yiä-Herttuala S. (2015). Angiopoietin-2 blocking antibodies reduce early atherosclerotic plaque development in mice. Atherosclerosis.

[47] Nykänen A.I., Krebs R., Saaristo A., Turunen P., Alitalo K., Ylä-Herttuala S., Koskinen P.K., Lemström K.B. (2003). Angiopoietin-1 protects against the development of cardiac allograft arteriosclerosis. Circulation.

[48] Pöss J., Fuernau G., Denks D., Desch S., Eitel I., de Waha S., Link A., Schuler G., Adams V., Böhm M. (2015). Angiopoietin-2 in acute myocardial infarction complicated by cardiogenic shock–a bio marker substudy of the IABP-SHOCK II-Trial. Eur. J. Heart Fail..

[49] Lee S., Lee C., Kang S., Park I., Kim Y.H., Kim S.K., Hong S.P., Bae H., He Y., Kubota Y. (2018). Angiopoietin-2 exacerbates cardiac hypoxia and inflammation after myocardial infarction. J. Clin. Investig..

[50] Gurnik S., Devraj K., Macas J., Yamaji M., Starke J., Scholz A., Sommer K., Di Tacchio M., Vutukuri R., Beck H. (2016). Angiopoietin-2 induced blood-brain barrier compromise and increased stroke size are rescued by VE-PTP-dependent restoration of Tie2 signaling. Acta Neuropathol..

[51] Golledge J., Clancy P., Maguire J., Lincz L., Koblar S., McEvoy M., Attia J., Levi C., Sturm J., Almeida O.P. (2014). Plasma angiopoietin-1 is lower after ischemic stroke and associated with major disability but not stroke incidence. Stroke.

[52] Ikegame Y., Yamashita K., Hayashi S., Yoshimura S., Nakashima S., Iwama T. (2010). Neutrophil elasetase inhibitor prevents ischemic brain damage via reduction of vasogenic edema. Hypertens. Res..

[53] Ikegame Y., Yamashita K., Hayashi S., Mizuno H., Tawada M., You F., Yamada K., Tanaka Y., Egashira Y., Nakashim S. (2011). Comparison of mesenchymal stem cells from adipose tissue and bone marrow for ischemic stroke therapy. Cytotherapy.

[54] Gevaert A.B., Boen J.R.A., Segers V., van Craenebroeck E.M. (2019). Heart failure with preserved ejection fraction: A review of cardiac and noncardiac pathophysiology. Front. Physiol..

[55] Chirinos J.A., Orlenko A., Zhao L., Basso M.D., Cvijic M.E., Li Z., Spires T.E., Yard M., Wang Z., Seiffert D.A. (2020). Multiple plasma biomarkers for risk stratification in patients with heart failure and preserved ejection fraction. J. Am. Coll. Cardiol..

[56] Wells Q.S., Gupta D.K., Smith J.G., Collins S.P., Storrow A.B., Ferguson J., Smith M.L., Pulley J.M., Collier S., Wang X. (2019). Accelerating biomarker discovery through electronic health records, automated bio-banking, and proteomics. J. Am. Coll. Cardiol..

[57] Cooke J.P., Meng S. (2020). Vascular regeneration in peripheral artery disease. Arterioscler. Thromb. Vasc. Biol..

[58] Rajesh G., Tongers J., Losordo D.W. (2009). Human studies of angiogenic gene therapy. Circ. Res..

[59] Nagy J.A., Benjamin L., Zeng H., Dvorak A.M., Dvorak H.F. (2008). Vascular permeability, vascular hyperpermeability and angiogenesis. Angiogenesis.

[60] Muona K., Mäkinen K., Hedman M., Manninen H., Ylä-Herttuala S. (2012). 10-year safety follow-up in patients with local VEGF gene transfer to ischemic lower limb. Gene Ther..

[61] Anisimov A., Tvorogov D., Alitalo A., Leppänen V.-M., An Y., Han E.C., Orsenigo F., Gaál E.I., Holopainen T., Koh Y.J. (2013). Vascular endothelial growth factor-angiopoietin chimera with improved properties for therapeutic angiogenesis. Circulation.

[62] Flugelman M.Y., Halak M., Yoffe B., Schneiderman J., Rubinstein C., Bloom A.-I., Weinmann E., Goldin I., Ginzburg V., Mayzler O. (2017). Phase 1b safety, two-dose study of MultiGeneAngio in patients with chronic critical limb ischemia. Mol. Ther..

[63] Findley C.M., Mitchell R.G., Duscha B.D., Annex B.H., Kontos C.D. (2008). Plasma levels of soluble Tie2 and vascular endothelial growth factor distinguish critical limb ischemia from intermittent claudication in patients with peripheral arterial disease. J. Am. Coll. Cardiol..

[64] Besnier M., Shantikumar S., Anwer M., Dixit P., Chamorro-Jorganes A., Sweaad W., Sala-Newby G., Madeddu P., Thomas A.C., Howard L. (2019). miR-15a/-16 inhibits angiogenesis by targeting the tie2 coding sequence: Therapeutic potential of a miR-15a/16 decoy system in limb ischemia. Mol. Ther. Nucleic Acids.

[65] Hayashi S., Morishita R., Nakamura S., Yamamoto K., Moriguchi A., Nagano T., Taiji M., Noguchi H., Takeshita S., Matsumoto K. (1999). Potential role of hepatocyte growth factor, in peripheral arterial disease: Downregulation of HGF in response to hypoxia in vascular cells. Circulation.

[66] Kobayashi H., DeBusk L.M., Babichev Y.O., Dumont D.J., Lin P.C. (2006). Hepatocyte growth factor mediates angiopoietin-induced smooth muscle cell recruitment. Blood.

[67] Chang F.C., Chiang W.C., Tsai M.H., Chou Y.H., Pan S.Y., Chang Y.T., Yeh Y.T., Chen C.K., Chu T.S., Wu K.D. (2014). Angiopoietin-2 induced arterial stiffness in CKD. J. Am. Soc. Nephrol..

[68] David S., Kümpers P., Hellpap J., Leitolf H., Haller H., Krelstein J.T. (2019). Angiopoietin2 and cardiovascular disease in dialysis and kidney transplantation. Am. J. Kidney Dis..

[69] David S., John S.G., Jefferies H.J., Sigrist M.K., Kümpers P., Kiekstein J.T., Haller H., McIntyre C.W. (2012). Angiopoietin-2 levels predict mortality in CKD patients. Nephrol. Dial. Transplant..

[70] Davis B., Dei Cas A., Long D.A., White K.E., Hayward A., Ku C.H., Woolf A.S., Bilous R., Viberti G., Gnudi L. (2007). Podocyte-specific expression of angiopoietin-2 causes proteinuria and apoptosis of glomerular endothelia. J. Am. Soc. Nephrol..

[71] World Health Organization (2020). Health Topics/Diabetes.

[72] Biessels G.J., Staekenborg S., Brunner E., Brayne C., Scheltens P. (2006). Risk of dementia in diabetes mellitus: A systematic review. Lancet Neurol..

[73] Lim H.S., Blann A.D., Chong A.Y., Freestone B., Lip G.Y.H. (2004). Plasma vascular endothelial growth factor, angiopoietin-1, and angiopoietin-2 in diabetes: Implications for cardiovascular risk and effects of multifactorial intervention. Diabetes Care.

[74] Tuuminen R., Haukka J., Loukovaara S. (2015). Poor glycemic control associates with high intravitreal Angiopoietin-2 levels in patients with diabetic retinopathy. Acta Opthalmol..

[75] Rangasamy S., Srinvasan R., Maestas J., McGuire P.G., Das A. (2011). A potential role for angiopoietin-2 in the regulation of the blood-retinal barrier in diabetic retinopathy. Investig. Ophthalmol. Vis. Sci..

[76] Hammers H.-P., Lin J., Wagner P., Feng Y., vom Hagen F., Krzizok T., Renner O., Breier G., Brownlee M., Deutsch U. (2004). Angiopoietin-2 causes pericyte dropout in the normal retina: Evidence for involvement in diabetic retinopathy. Diabetes.

[77] Cho C.H., Kammerer R.A., Lee H.J., Steinmetz M.O., Ryu Y.S., Lee S.H., Yasunaga K., Kim K.-T., Kim I., Choi H.H. (2004). COMP-Ang1: A designed Angiopoietin-1 variant with nonleaky angiogenic activity. Proc. Natl. Acad. Sci. USA.

[78] Cahoon J.M., Rai R.R., Carroll L.S., Uehara H., Zhang X., O’Neil C.L., Medina R.J., Das S.K., Muddana S.K., Olson P.R. (2015). Intravitreal AAV2.COMP-Ang1 Prevents Neurovascular Degeneration in a Murine Model of Diabetic Retinopathy. Diabetes.

[79] Campochiaro P.A., Khanani A., Singer M., Patel S., Boyer D., Dugel P., Kherani S., Withers B., Gambino L., Peters K. (2016). Enhanced benefit in diabetic macular edema from AKB-9778 Tie2 activation combined with vascular endothelial growth factor suppression. Ophthalmology.

[80] Dessapt-Baradez C., Woolf A.S., White K.E., Pan J., Huang J.L., Hayward A.A., Price K.L., Kolatsi-Joannou M., Locatelli M., Diennet M. (2014). Targeted glomerular angiopoietin-1 therapy for early diabetic kidney disease. J. Am. Soc. Nephrol..

[81] Yamamoto K., Maeshima Y., Kitayama H., Kitamura S., Takazawa Y., Sugiyama H., Yamasaki Y., Makino H. (2004). Tumstatin peptide, an inhibitor of angiogenesis, prevents glomerular hypertrophy in the early stage of diabetic nephropathy. Diabetes.

[82] Carota I.A., Kenig-Kozlovsky Y., Onay T., Scott R., Thomson B.R., Souma T., Bartlett C.B., Li Y., Procissi D., Ramirez V. (2019). Targeting VE-PTP phosphatase protects the kidney from diabetic injury. J. Exp. Med..

[83] Armstrong G.C., Boulton A.J.M., Bus S.A. (2017). Diabetic foot ulcers and their recurrence. N. Engl. J. Med..

[84] Prompers L., Schaper N., Apelqvist J., Edmonds M., Jude E., Mauricio D., Uccioli L., Urbancic V., Bakker K., Holstein P. (2008). Prediction of outcome in individuals with diabetic foot ulcer: Focus on the differences between individuals with and without peripheral arterial disease. The EURODIALE Study. Diabetologia.

[85] Eming S.A., Martin P., Tomic-Canic M. (2014). Wound repair and regeneration: Mechanisms, signaling, and translation. Sci. Transl. Med..

[86] Everett E., Mathioudakis N. (2008). Update on management of diabetic foot ulcer. Ann. N. Y. Acad. Sci..

[87] Kämpfer H., Pfeilschifter J., Frank S. (2001). Expressional regulation of angiopoietin-1 and -2 and tie-1 and tie-2 receptor tyrosine kinases during cutaneous wound healing: A comparative study of normal and impaired repair. Lab. Investig..

[88] Cho C.-H., Sung H.-K., Kim K.-T., Cheon H.G., Oh G.T., Hong H.J., Yoo O.-J., Koh G.Y. (2006). COMP-angiopoietin-1 promotes wound healing through enhanced angiogenesis, lymphangiogenesis, and blood flow in a diabetic mouse model. Proc. Natl. Acad. Sci. USA.

[89] Xiao Y., Reis L.A., Feric N., Knee E.J., Gu J., Cao S., Laschinger C., Londono C., Antolovichi J., McGuigan A.P. (2016). Diabetic wound regeneration using peptide-modified hydrogels to target re-epithelialization. Proc. Natl. Acad. Sci. USA.

[90] Miklas J.W., Dallabrida S.M., Reis L.A., Ismail N., Rupnick M., Radisic M. (2013). QHREDGS enhances tube formation, metabolism and survival of endothelial cells in collagen-chitosan hydrogels. PLoS ONE.

[91] Alzheimer A. (1907). Uber eine eigenartig Erkrankung der Hirnrinde. Allg. Z. Psychiatrie Psych. Ger. Med..

[92] Roth M. (1995). The natural history of mental disorder in old age. J. Ment. Sci..

[93] Selkoe D.J. (1991). The molecular pathology of Alzheimer’s disease. Neuron.

[94] Harman D. (2006). Alzheimer’s disease pathogenesis: Role of aging. Ann. N. Y. Acad. Sci..

[95] De La Torre J.C. (2004). Is Alzheimer’s disease a neurodegenerative or a vascular disorder? Data, dogma, and dialectics. Lancet Neurol..

[96] Hachinsky V. (2008). Shifts in thinking about dementia. JAMA.

[97] Hayashi S., Sato N., Yamamoto A., Ikegame Y., Nakashima S., Ogihara T., Morishita R. (2009). Alzheimer disease-associated peptide, amyloid beta 40, inhibits vascular regeneration with induction of endothelial autophagy. Arterioscler. Thromb. Vasc. Biol..

[98] Raman M.R., Himali J.J., Conner S.C., DeCarli C., Vasan R.S., Beiser A.S., Seshadri S., Maillard P., Satizabal C.L. (2018). Circulating vascular growth factor and magnetic resonance imaging markers of small vessel disease and atrophy in middle-aged adults. Stroke.

[99] Yamazaki Y., Zhao N., Caulfield T.R., Liu C., Bu G. (2019). Apolipoprotein E and Alzheimer’s disease: Pathobiology and targeting strategies. Nat. Rev. Neurol..

[100] Schreitmuller B., Leyhe T., Stransky E., Köhler N., Laske C. (2012). Elevated angiopoietin-1 serum levels in patients with Alzheimer’s disease. Int. J. Alzheimers Dis..

[101] Whitty C.J.M., Watt F.M. (2020). Map clusters of diseases to tackle multimorbidity. Nature.

[102] Fauci A.S., Lana H.C., Redfield R.R. (2020). Covid-19-Navigating the uncharted. N. Engl. J. Med..

[103] Richardson S., Hirsch J.S., Narasimhan M., Crawford J.M., McGinn T., Davidson K.W., Barnaby D., Becker L.B., Chelico J.D., Cohen S.L. (2020). Presenting characteristics, comorbidities, and outcomes among 5700 patients hospitalized with COVID-19 in the New York city area. JAMA.

[104] Matthay M.A., Zemans R.L., Zimmerman G.A., Arabi Y.M., Beitler J.R., Mercat A., Herridge M., Randolph A.G., Calfee C.S. (2019). Acute respiratory distress syndrome. Nat. Rev. Dis. Primers.

[105] Davis S., Papadopoulos N., Aldrich T.H., Maisonpierre P.C., Huang T., Kovac L., Xu A., Leidich R., Radziejewska E., Rafique A. (2003). Angiopoietins have distinct modular domains essential for receptor binding, dimerization and superclustering. Nat. Struct. Biol..

[106] Liu P., Ryczko M., Xie X., Baardsnes J., Lord-Dufour S., Duroche Y., Hicks E.A., Taiyab A., Sheardown H., Quaggin S. (2020). New soluble angiopoietin analog of Hepta-Ang1 prevents pathological vascular leakage. Biotechnol. Bioeng..

